# Change Detection of Remote Sensing Images Based on Attention Mechanism

**DOI:** 10.1155/2020/6430627

**Published:** 2020-08-25

**Authors:** Long Chen, Dezheng Zhang, Peng Li, Peng Lv

**Affiliations:** ^1^School of Computer and Communication Engineering, University of Science and Technology Beijing (USTB), No. 30 Xueyuan Road, Haidian District, Beijing 100083, China; ^2^Beijing Key Laboratory of Knowledge Engineering for Materials Science, Beijing, China

## Abstract

In recent years, image processing methods based on convolutional neural networks (CNNs) have achieved very good results. At the same time, many branch techniques have been proposed to improve accuracy. Aiming at the change detection task of remote sensing images, we propose a new network based on U-Net in this paper. The attention mechanism is cleverly applied in the change detection task, and the data-dependent upsampling (DUpsampling) method is used at the same time, so that the network shows improvement in accuracy, and the calculation amount is greatly reduced. The experimental results show that, in the two-phase images of Yinchuan City, the proposed network has a better antinoise ability and can avoid false detection to a certain extent.

## 1. Introduction

Change detection in remote sensing images is a critical and challenging task, and its specific work refers to the quantitative analysis of multiple temporal remote sensing images for the same target area, determining the features and scope of surface changes and detecting the changed and unchanged parts [1]. Remote sensing image change detection is utilized to detect illegal buildings, water area supervision, natural disaster assessment, urban planning expansion research, and military reconnaissance [2].

Because of the increasing amount of data from remote sensing images and the increasing demand in this direction, manual comparison and analysis of the change area appear time-consuming and laborious. Due to factors such as seasons and solar illumination, imaging styles of different phases have huge differences [3], which make it difficult to solve the change detection task by computer vision.

**Figure 1:**
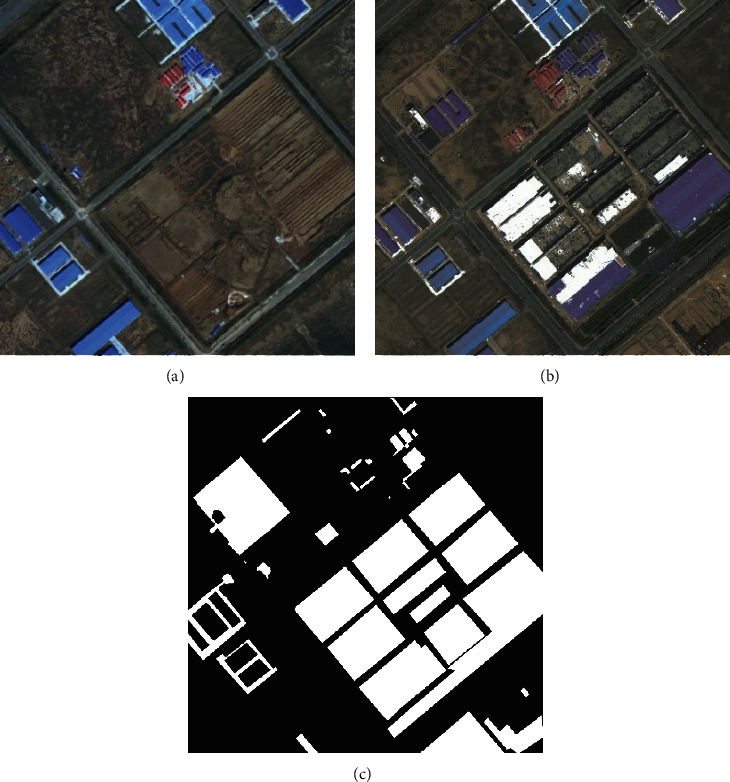
Example of remote sensing image change detection.

Change detection is a unique task for remote sensing image processing, which can be regarded as a dichotomy problem of a region changing or not, as shown in Figure 1.  Figure 1(a) shows the remote sensing images of a certain region of Yinchuan City in 2015, Figure 1(b) shows the remote sensing images of this region in 2017, and Figure 1(c) shows the change label of this region, where black indicates that the location has not changed and white indicates that the location has changed. The task of change detection is to identify the changing areas in different phases.

General change detection methods are mainly based on autoencoders and feature extraction through the full connection between neurons [4]. But in fact, change detection can flexibly apply the method of semantic segmentation and extract features by convolution. CNNs have led the field in image processing since AlexNet [5] won the championship in 2012. With the advent of networks such as FCN [6], U-Net [7], and SegNet [8], the baseline effect in this field is getting better and better. However, due to the characteristics of the change detection task, the above-mentioned excellent networks often cannot exert the best results.

In this paper, our task is to solve the change detection in three districts of Yinchuan City. After a detailed analysis of this task, we found that the volume of the changed part is much smaller than that of the unchanged part. There is a greater degree of positive and negative sample imbalance problem. To this end, we propose a new network based on U-Net. The network consists of encoder and decoder. Based on the residual attention model [9], we proposed a new attention mask structure for feature extraction, and a new encoder structure is also proposed in order to better perceive the changing area. By generating an attention mask, the model can pay more attention to the regions with obvious changes and improve the antinoise ability of the model. In the decoder stage, a data-dependent upsampling method (DUpsampling) [10] is used to replace the general upsampling method. The new upsampling method can be applied to smaller-resolution feature maps, which greatly reduces the computational complexity. At the same time, we propose a new loss function for the network, and the initial values of different loss functions are used to balance its impact on network training and reduce the impact of sample imbalance.

## 2. Related Work

### 2.1. Convolutional Neural Network

The achievement of today's success in image processing largely depends on the CNNs [11]. The essence of CNNs is a multilayer perceptron. The network structure includes a convolutional layer, a downsampling layer, and a fully connected layer. The reason for its success is local connection and weight sharing method [12]. Reducing the number of weight makes the network easy to optimize and reduces the complexity of the model, which reduces the risk of overfitting. The earliest CNNs are time-delayed neural networks [13] and Lenet-5 [14]. After AlexNet won the champion of ILSVRC [15] in 2012, thanks to the support of GPU computing cluster, deep CNNs such as ZFNet [16], VGGNet [17], and GoogLeNet [18] became the winning algorithm of ILSVRC for many times. But at the same time, CNNs fail to converge with the deepening of network layers. ResNet [19] proposes the mechanism of residual learning, making the network easy to converge while getting deeper. However, the original CNN receptive field is small, and it cannot sense the neighborhood information well. Enlarging the receptive field will lead to a large increase in computing resources. At the same time, CNN's fully connected mode is too redundant and inefficient.

### 2.2. Attention Mechanism

Mnih et al. [20] confirmed the effectiveness of attention mechanism. Attention is generally classified into two types: one is top-down conscious attention, called focus attention. The other is bottom-up unconscious attention, called saliency-based attention. Focus attention refers to the attention that has a predetermined purpose and focuses on a certain object actively and consciously [21, 22]. Saliency-based attention is also called stimulation-based attention [23]. Wang et al. [9] proposed a method to solve the problem of image classification by using attention residual learning.

### 2.3. Encoder-Decoder Architectures

Since FCN was proposed, people have been trying to use FCN to improve the accuracy of pixel-level prediction. On the one hand, people start with the atrous convolution [24, 25], which needs more complex operations, and on the other hand, people use encoder-decoder architectures. The most significant feature of encoder-decoder architectures is the ability to complete end-to-end learning. U-Net improves on the FCN framework by connecting codecs with skip connections to improve the effect. SegNet records the location of the maximum value during the maximum pooling operation of the encoder part and then realizes nonlinear upsampling through the corresponding pooling index in the decoder. DeepLab V3 [26] uses the ASPP structure to expand the receptive field, mining context information, and the improved Xception module to reduce the number of parameters and achieve the best effect of the current semantic segmentation network. Tian et al. [27] proposed a data-dependent upsampling method, which enables the encoder to sample down to the bottom layer and improve the accuracy by fusing features of different layers.

### 2.4. Change Detection

Remote sensing image change detection can greatly improve land utilization and contribute to urban planning and expansion. In the first decade of this century, CNN was rarely used in the field of change detection. Carincotte et al. [28] used a fuzzy hidden Markov chain algorithm to avoid a large number of false changes and missed detections caused by threshold segmentation. Liu et al. [29] used the stacked restricted Boltzmann machine to analyze the differential images between multiphase SAR images and classified the neighborhood features of the two-phase images. By using the deep learning algorithm, the images were classified pixel by pixel to achieve the purpose of change detection. In recent years, CNNs began to be implemented in this field. Desclee et al. [30] proposed an object-oriented forest vegetation change detection method, which firstly segments multitemporal high-resolution remote sensing images and then, based on the hypothesis chi-square test, identifies outliers of statistical differences in reflectivity and marks corresponding objects as changes. Qing et al. [31] applied Faster R-CNN to this field, greatly reducing the false changes of detection results. Ma et al. proposed a network based on multigrained cascade forest and multiscale fusion, so that the network can select image blocks of different sizes as input, thereby learning more image features [32]. Dong et al. designed a “Siamese samples” convolutional neural network to learn the semantic difference between changed and unchanged pixels [33].

The change detection in Yinchuan area includes many different landforms. Considering the particularity of this region, our method adds the attention module in the feature extraction stage and uses U-Net's skip connection to further reduce the loss of information from upsampling and downsampling, it also uses DUpsampling to accelerate the upsampling process, while facilitating subsequent feature fusion. As a result, our model can avoid many false detections, while ensuring accuracy without occupying too many computing resources.

## 3. Our Approach

In this section, we first introduce the network we proposed and then elaborate on each functional module of the network, and we also propose a new loss function based on the problem we meet to improve the accuracy of the model. It is noteworthy that, different from the current mainstream of change detection method based on the convolution neural network, we improve the residual attention mechanism, and we proposed a new way to generate attention mask and apply the mask to change detection task. At the same time, the DUpsampling method is used to reduce the loss of computing resources. Finally, we noticed the impact of the initial size of the loss function on the network performance and proposed a new loss function, which achieved good results.

### 3.1. Network Architecture

**Figure 2:**
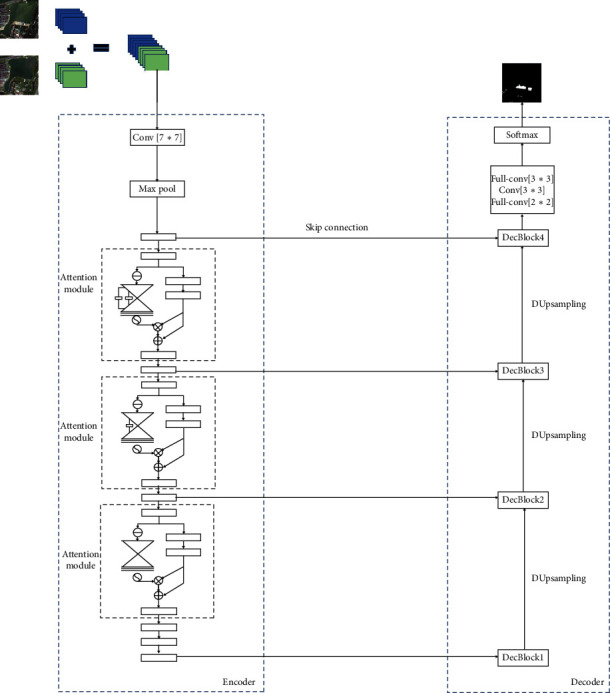
The framework of our proposed network.

In this paper, we propose a new network based on U-Net, as shown in Figure 2. Like U-Net, the network is divided into encoder and decoder. The encoder downsamples the input to extract the features, while the decoder upsamples the input to restore the resolution. The network uses a six-channel matrix superimposed on two pictures of different phases in the same area as input to the encoder. The encoder takes ResNet50 as the trunk and adds the attention modules. The attention module consists of trunk branch and mask branch, which perform feature extraction and mask generation on the input, respectively. The attention mask subtracts the three channels of two RGB images as the input and outputs a feature map with attention weight to highlight the key areas of feature extraction. Then residuals are performed on feature maps of mask branch and trunk branch. The DUpsampling method is used to replace the conventional bilinear upsampling on the decoder, which avoids the computation and memory footprint caused by reducing step size (such as DeepLab V3) of the decoder. The decoder maintains the original network structure of U-Net. There are skip connections between the encoder and the decoder to combine the features of the corresponding encoding during decoding.

### 3.2. Functional Modules

#### 3.2.1. Encoder

**Figure 3:**
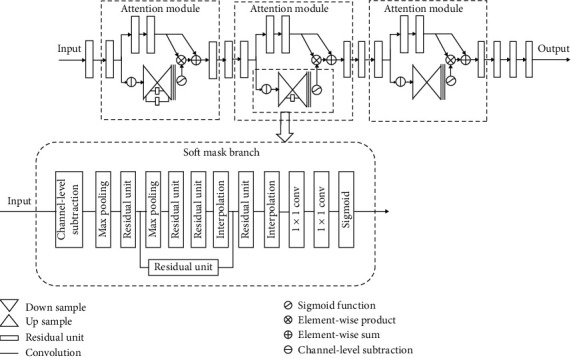
The framework with our proposed encoder which includes three attention modules and some residual units.

In order to improve the accuracy of the network, different from the traditional autoencoder network, we choose to deepen the network depth, thus introducing ResNet50. Its unique residual learning method can avoid the problem of unable to converge due to gradient explosion or gradient disappearance in deep network. In the encoder stage, ResNet50 as the backbone, and the residual attention mechanism is added to form a new encoder structure to guide the network to focus on areas with significant changes and improve the network's antinoise ability. The encoder includes three attention modules as shown in Figure 3. Each attention module is divided into mask branch and trunk branch. Trunk branch performs feature extraction just like normal convolutional neural networks. Mask branch is responsible for generating attention weights for input features, and finally, residuals are performed on feature maps of mask branch and trunk branch. The formula is as follows:

(1) $$\begin{array}{c} H_{i,c}\left(x\right)=\left(1+M_{i,c}\left(x\right)\right)*F_{i,c}\left(x\right), \end{array}$$

where *F*_*i*,*c*_(*x*) represents the features generated by the deep convolutional network, *M*_*i*,*c*_(*x*) represents the output of the mask branch, and the value range of *M*_*i*,*c*_(*x*) is [0,1]. When it approaches 0, *H*_*i*,*c*_(*x*)is approximately the original feature*F*_*i*,*c*_(*x*). The detailed structure is illustrated in Table 1.

#### 3.2.2. Attention Module

Because change detection requires comparing information of different phases in the same region, we propose a new architecture of the attention modules, which is different from semantic segmentation task when generating attention weight. In the mask branch, we subtract each channel of the original two feature maps to generate a new three-channel feature map. This step is called the channel-level subtraction. The new feature map is used to generate the attention mask and then put it into the mask branch for upsampling and downsampling and convolution, and the purpose of this step is to downsample to low resolution so that we can get strong semantic information. Each mask branch has a different number of residual units between upsampling and downsampling as skip connections to capture attention information at different scales. Each time pass the residual unit connected to the attention modules, and the size of the feature map is reduced. The architecture is displayed in Table 1.

#### 3.2.3. Decoder

We keep the decoder structure of U-Net. The proposed network uses DUpsampling in the upsampling phase to replace the original bilinear upsampling procedure. The bilinear upsampling method does not take into account the correlation between the predicted pixels. DUsampling uses the redundancy of segmentation labels to produce accurate segmentation results through the rough features generated by the encoder. And the encoder structure does not need to continue to excessively reduce the resolution of the feature map, thereby reducing the calculation time and memory usage. An important discovery is that the label of images is not independently and uniformly distributed, and the structural information it contains is related, so the label can be compressed without causing too much loss. So we compress the label first and split label into multiple grids, and each grid size is *t∗t* (*t* is the image size ratio, such as 16 and 32), and then we reshape the content of each grid into a vector *v*, then compress *v* into *x*, and stack *x* to get compressed labels. Formally, we have

(2) $$\begin{array}{c} x=Pv, \end{array}$$

(3) $$\begin{array}{c} \tilde{v}=Wx. \end{array}$$

Linearly map *v* to *x* through *P*, and *W* is the inverse mapping matrix, which is the reconstruction matrix. Through the following formula to minimize the reconstruction error and optimize through SGD iteration, PCA [34] can be used to obtain the closed solutions *P* and *W*. Formally,

(4) $$\begin{array}{c} P^{*},W^{*}=\underset{P,W}{\arg\min}\sum_{v}v-{\tilde{v}}^{2}\\ =\underset{P,W}{\arg\min}\sum_{v}v-WPv^{2}. \end{array}$$

**Figure 4:**
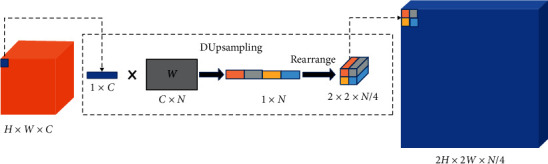
The specific process of DUpsampling. *W* is the reconstruction matrix, and there are three DUpsamplings in our network.

When we get the reconstructed matrix *W*, *W* is the parameter of the convolution kernel, which can complete the upsampling procedure, as shown in Figure 4.

At the same time, adaptive temperature softmax is introduced because the DUpsampling method may be calculated based on the one-hot label, so that the probability distribution is relatively smooth, resulting in too slow or even difficult convergence of loss in training [35], as shown in the following equation:

(5) $$\begin{array}{c} \text{sotfmax }z_{i}=\frac{\exp\left(z_{i}/T\right)}{\sum_{j}\exp\left(z_{i}/T\right)}. \end{array}$$

*T* can be learned automatically by the backpropagation algorithm without tuning. There is a skip connection between the encoder and the decoder so that the decoder can obtain the feature information in the encoder and reduce the loss of information in the decoding procedure. The decoder part contains four decoders, whose input includes the encoder map from skipping connection and the output of the previous layer. Through the upsampling procedure, the size of the feature map is doubled. Finally, two full convolutional layers and one convolutional layer are added, so that the size is enlarged again to achieve the effect of end-to-end training, as shown in Figure 2.

#### 3.2.4. Loss

The two images of the change detection mission in the three districts of Yinchuan were taken in 2015 and 2017. When we look at the two phases of a total of 924 images and found that the volume of the changed part is much smaller than that of the unchanged part, change detection is essentially a binary classification problem, namely, one regional changes or not. There is a greater degree of positive and negative samples by imbalance problem. Therefore, we introduced the focal loss function [36], which can effectively solve the imbalance of positive and negative samples, as part of the loss function. Formally,

(6) $$\begin{array}{c} FL\left(p_{t}\right)=-\alpha_{t}{\left(1-p_{t}\right)}^{\gamma }\log\left(p_{t}\right), \end{array}$$

where *p*_*t*_ is the classification probability of different categories and *γ* and *α*_*t*_ are fixed values. Here, *γ* = 2 and *α*_*t*_ = 0.25. Combined with the binary crossover entropy loss function *L*_*BCE*_, which is commonly used in this task, combining the above two loss functions, we proposed a new loss function. First of all, we noticed that different types of loss functions have different initial values. Therefore, if we simply weighted the two loss functions, the loss function with a large initial value would dominate the loss function with a small initial value. We use the initial values of the two loss functions obtained in the first iteration to balance:

(7) $$\begin{array}{c} \text{loss}=\frac{c_{1}}{c_{1}+c_{2}}FL\left(p_{t}\right)+\frac{c_{2}}{c_{1}+c_{2}}L_{BCE}, \end{array}$$

where *c*_1_ is the initial value of *L*_*BCE*_ and *c*_2_ is the initial value of focal loss. Experiments show that *c*_1_ is much larger than *c*_2_, often several times larger than *c*_2_, so that different loss functions will not distinguish the primary and secondary relationship due to different sizes. And then we add the two loss functions together to get the final loss, and with *α* approximating 0.3, our network will achieve the best results. The loss function can be formulated as follows:

(8) $$\begin{array}{c} \text{loss}=\alpha \frac{c_{1}}{c_{1}+c_{2}}FL\left(p_{t}\right)+\left(1-\alpha \right)\frac{c_{2}}{c_{1}+c_{2}}L_{BCE}. \end{array}$$

## 4. Experiments

In this section, we first explain the datasets we used and the criteria we used to evaluate the effect of the method and then introduce the relevant experimental details. At the same time, we performed ablation experiments on the proposed loss function, verified the effectiveness of our loss function, and found the optimal value of *α*. Finally, we compare the effect of the current mainstream network with our network.

### 4.1. Datasets

**Figure 5:**
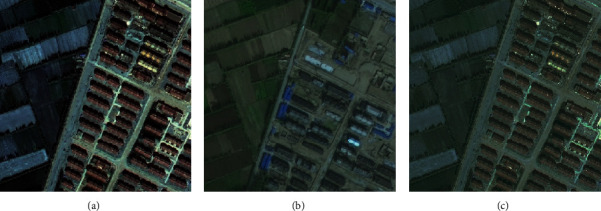
Effect before and after histogram matching. (a) The original images of 2017, (b) the original images of 2015, and (c) the processed image of 2017.

In our experiment, the datasets came from Gaofen-2 (Gf-2) satellite including Xixia District, Xingqing District, and Jinfeng District of Yinchuan City, known as Yinchuan three districts. The ground resolution of Gf-2 is 1 meter, indicating that per pixel represents one square meter. The image has four channels, namely, RGB channels and near-infrared channel. Since the year 2015 image has only RGB channels, we take RGB channels in both phases. The images we used have been irradiated and registered in absolute terms, but not in relative terms. Therefore, we first performed histogram matching on the image to reduce the radiation difference between the two images, as shown in Figure 5.

Due to the particularity of change detection, we need to select the original datasets and eliminate the regions with no change or those with little change. Finally, 924 images from Gf-2 satellite were selected, covering different areas such as towns, agriculture, and industrial areas. Each image size was 512*∗*512.The datasets were divided into training set, verification set, and test set at 7 : 2  : 1, with 647, 184, and 92 pictures, respectively. Because of the small amount of dataset, U-Net, which has relatively loose requirements on dataset size, was chosen as the basic framework at the beginning of designing the network.

### 4.2. Evaluation Criteria

In our experiment, accuracy and *F*1 value are used as the accuracy evaluation standard, and FLOPS is used as the efficiency evaluation standard. The calculation formula of accuracy is as follows:

(9) $$\begin{array}{c} \text{accuracy}=\frac{\left(\text{TP}+\text{TN}\right)}{\left(\text{P}+\text{N}\right)}, \end{array}$$

where TP is the number of pixels with a positive detection number representing actual changes that are correctly detected and FP is the number of pixels with false detection number representing actual unchanged that are erroneously detected as changes. TP and FP correspond to TN and FN. TN is the number of pixels that have not actually changed but detected as changed and FN is the number of pixels that have actually changed but detected as not changed. P = TP + FN and N=FP + TN. The calculation formulas of precision and recall are as follows:

(10) $$\begin{array}{c} \text{precision}=\frac{\text{TP}}{\text{TP}+\text{FP}}, \end{array}$$

(11) $$\begin{array}{c} \text{recall}=\frac{\text{TP}}{\text{TP}+\text{FN}}. \end{array}$$

The precision represents the proportion of correctly detected change areas in the predicted results. The higher the precision, the less the false changes and noise, and recall represents the proportion of correctly detected change areas in the actual change areas. The higher the recall, the better the coverage of change detection results to the true change results. On the basis of these two important indicators, we calculated the weighted harmonic average of precision and recall. As a comprehensive indicator, *F*1 value is used here:

(12) $$\begin{array}{c} F1=\frac{2\times \text{precision}\times \text{recall}}{\text{precision}+\text{recall}}. \end{array}$$

FLOPS is an abbreviation of floating-point operations per second. It is often used to estimate the performance of a network.

### 4.3. Experimental Details

There are a number of areas in our datasets that have not changed, so we filter the datasets. The size of each picture is 512*∗*512, which is about 250,000 pixels. We consider pictures with a total number of changed pixels less than 1000 as unchanged and removed. In order to enhance the generalization of the network, after histogram matching of the images and reducing the imaging difference, we carry out random geometric transformation and color adjustment of the images in a small range and normalize each channel value of the processed remote sensing images, so that the network could rapidly converge. We use the Adam optimizer [37] to train 1000 epochs on the network, and the initial learning rate is designed to be 1 *e* − 3. After each epoch, the learning rate dropped and *α* in the loss function is set to 0.3. We observe that our networks generally converged after 200 epochs.

### 4.4. Loss Function Ablation Experiments

To prove the effectiveness of our loss function, we performed two experiments in this section. First, use formulas (7) and (13) as loss functions, respectively, to compare the effect of initial value balance on experimental results:

(13) $$\begin{array}{c} \text{loss}=FL\left(p_{t}\right)+L_{BCE}, \end{array}$$

As mentioned above, the initial value of *L*_*BCE*_ is much larger than the focal loss, so during the training process, *L*_*BCE*_ will occupy the dominant position. The datasets are mentioned in Section 4.1. The experimental details are mentioned in Section 4.3. The initial learning rate is set to 1 *e*−3. The Adam optimizer is used to train 1000 epochs on multiple networks. The experimental results are shown in Table 2. At the same time, the network has significantly improved the convergence speed after the initial value balance.

After confirming the effectiveness of the initial value balance, we began to explore the optimal value of *α*. When *α*=0, it means that only *L*_*BCE*_ works, and when *α*=1, only focal loss works. The results of the comparative experiments are as follows.

**Figure 6:**
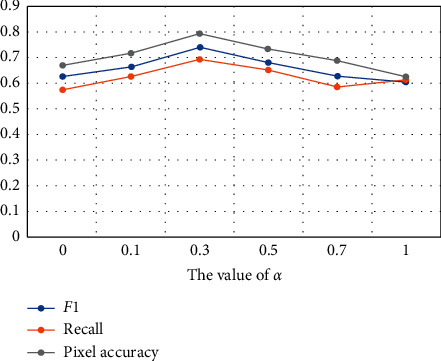
The effect of different values of *α* on the experimental results. It proves that our method has an optimal solution when *α*=0.3.

It can be seen from Figure 6 and Table 3 that, for our problem, when *α*=0.3, the network has the best performance. The reason may be that the imbalance of positive and negative samples was improved after the introduction of residual attention mechanism, so the effect of focal loss was not optimal. In the following experiments, we set *α* to 0.3 by default and compared it with other networks.

### 4.5. Experimental Results

**Figure 7:**
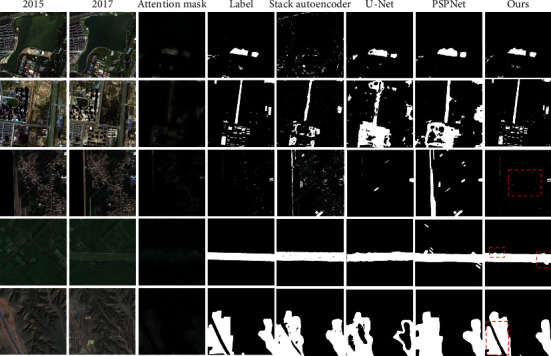
The attention mask and extraction results of the stack autoencoder, U-Net, PSPNet, and ours.

We select three mainstream networks to compare with our network on our datasets (mentioned in Section 4.1), including the stack autoencoder based on deep confidence network [38], U-Net, and PSPNet [39]. All networks are trained and tested on our datasets without using pretrained models. As shown in Figure 7, from left to right are the year 2015 images, the year 2017 images, attention mask, labels, and the results of different networks. The images on display include different scenes of cities, towns, fields, and bare grounds. As shown in the figure, our model is superior to other models in most cases.  Table 4 shows the performance of the above networks and ours on *F*1 value, recall, and accuracy. As shown in the table, the networks based on the convolution are superior to the stack autoencoder in our datasets, and PSPNet has a strong ability of context information so that it performs better than ordinary U-Net, and our network is 1.9% higher than PSPNet in *F*1 and 5.1% higher in accuracy. The performance of *F*1 and accuracy verifies that our network and the proposed loss functions are effective. At the same time, the FLOPS value of our network is 4.5 × 10^9^, which is far lower than PSPNet and U-Net. It shows that our method has high efficiency while ensuring accuracy and reduces waste of computing resources. It is worth mentioning that in the attention modules, our network will subtract the corresponding channels of input to generate the attention mask. In the figure, we will visualize the attention mask, and the attention mask will optimize the network training process, thus greatly improving the antinoise ability of our network. Thanks to the attention mask, our network can avoid many false detections, such as the third set of pictures. We analyzed in detail that our task for two-phase imaging style difference is huge, so we need to do histogram match in the pretreatment stage, but after the match, color distortion will happen, for example, the third group pictures had dark green fields into a dark purple, which greatly affected the judgment of the network, excellent network like PSPNet is also unable to avoid this problem. In the fifth group pictures, we can see that the attention mask divides the left variable area into two parts, and our network can make correct judgments, while most other networks are unable to. We also observe some disadvantages from the experiment, such as the fourth group experiment, mask only focuses on the middle of the road section and ignores the rest of the change, so the module cannot detect the change of the upper left corner buildings like PSPNet; hence, generating accurate attention mask is the key to the success of our network. At the same time, because we adopt the DUpsampling method, different from the ordinary method, the training speed will be very fast. This experiment only took about 30 hours to train on the latest GPU. At the same time, it is also convenient for feature fusion of different output layers [26] to improve the accuracy. Experiments show that the accuracy of our proposed network is greatly improved compared with the baseline. In complex scenes such as densely built towns, thanks to the attention mechanism, our network can reduce false detection and missed detection and show a better effect.

## 5. Conclusion

In this paper, we propose a new network based on U-Net by combining the skip connection structure with the advanced residual attention mechanism and the DUpsampling method, and we also propose a new loss function suitable for application scenarios. Our network is applied to Yinchuan change detection task, and experiments show that compared with the current change detection methods, our network and loss function have improved accuracy and *F*1 value without wasting excessive computing resources. At the same time, our method has a strong antinoise ability and certain robustness which can better solve the change detection task of Yinchuan City.

## Figures and Tables

**Table 1:** The architecture details for our encoder.

Layer	Output size	Encoder
Conv1	512 × 512	7 *×* 7, 64, stride 2

Max pooling	256 × 256	3 *×* 3 stride 2

Residual unit	256 × 256	$\left(\begin{array}{c} \begin{array}{c} 1\times 1,64\\ 3\times 3,64 \end{array}\\ 1\times 1,256 \end{array}\right)\times 1$

Attention module	256 × 256	Attention *×* 1

Residual unit	128 × 128	$\left(\begin{array}{c} \begin{array}{c} 1\times 1,128\\ 3\times 3,128 \end{array}\\ 1\times 1,512 \end{array}\right)\times 1$

Attention module	128 × 128	Attention *×* 1

Residual unit	64 × 64	$\left(\begin{array}{c} \begin{array}{c} 1\times 1,256\\ 3\times 3,256 \end{array}\\ 1\times 1,1024 \end{array}\right)\times 1$

Attention module	64 × 64	Attention *×* 1

Residual unit	32 × 32	$\left(\begin{array}{c} \begin{array}{c} 1\times 1,64\\ 3\times 3,64 \end{array}\\ 1\times 1,256 \end{array}\right)\times 3$

**Table 2:** Comparison of the effects of two loss functions on the network.

Method	*F*1	Recall	Pixel accuracy	Convergence epoch
Formula (7)	**0.615**	**0.608**	**0.693**	**340**
Formula (13)	0.575	0.554	0.574	570

**Table 3:** Comparison of *F*1, recall values, and pixel accuracy values with different values of *α*.

*α*	*F*1	Recall	Pixel accuracy
0	0.628	0.576	0.672
0.1	0.666	0.629	0.717
**0.3**	**0.743**	**0.697**	**0.796**
0.5	0.686	0.653	0.735
0.7	0.626	0.584	0.694
1	0.607	0.617	0.627

**Table 4:** Comparison of *F*1, recall values, and FLOPS values with the stack autoencoder, U-Net, PSPNet, and ours on the test datasets.

Module	*F*1	Recall	Pixel accuracy	FLOPS × 10^9^
Stack autoencoder	0.463	0.391	0.568	3.2
U-Net	0.689	0.691	0.687	5.6
PSPNet	0.724	0.704	0.745	11.2
Ours	0.743	0.697	0.796	4.5

## Data Availability

The data used to support the findings of this study are available from the corresponding author upon request, but for study only, not for commercial use.
